# Relationship between bony tunnel and knee function in patients after patellar dislocation triple surgeries—a CT-based study

**DOI:** 10.1038/srep41360

**Published:** 2017-01-25

**Authors:** Le Qin, Mei Li, Weiwu Yao, Ji Shen

**Affiliations:** 1 Department of Radiology, Shanghai Jiao Tong University Affiliated Sixth People’s Hospital, Shanghai, 200233, China; 2 Department of Orthopedics, Shanghai Jiao Tong University Affiliated Sixth People’s Hospital, Shanghai, 200233, China

## Abstract

We aimed to assess the CT-based bony tunnel valuations and their correlation with knee function after patellar dislocation triple surgeries. A retrospective study was performed on 66 patients (70 knees) who underwent patellar dislocation triple surgeries. The surgery was MPFL reconstruction primarily, combined with lateral retinaculum release and tibial tubercle osteotomy. CT examinations were performed to determine the femoral tunnel position, along with the patellar and femoral tunnel width 3 days and more than 1 year after operation for follow-up. Functional evaluation based on Kujala and Lysholm scores was also implemented. We compared tunnel width of the first and last examinations and correlated femoral tunnel position of the last examination with knee function. At the last follow-up, femoral tunnel position in the anterior-posterior direction was moderately correlated with knee function. Femoral tunnel position in the proximal-distal direction was not associated with postoperative knee function. Patellar and femoral tunnel width increased significantly at the last follow-up. However, no significant functional difference was found between patients with and without femoral tunnel enlargement. Our results suggested that the tunnel malposition in anterior-posterior position based on CT was related to impaired knee function during the follow-ups.

Patellar instability or dislocation is a common cause of anterior knee pain and acute traumatic hematoma in young patients^1^,^2^. Risk factors for patellar instability include increased Tibial Tubercle-Trochlear Groove (TT-TG) distance, trochlear dysplasia, patellar alta, patellar tilt, etc^3^. Currently, medial patellofemoral ligament (MPFL) is considered as the most important structure for static stabilization, providing 50–60% of the restraint strength to prevent lateral patellar dislocation^4^,^5^. For this reason, MPFL reconstruction is broadly used to treat patellar dislocation. It is a safe and valid modality that leads to ideal clinical outcomes^6^. MPFL reconstruction could be adopted simultaneously with lateral retinaculum release and tibial tubercle osteotomy^7^.

Postoperative imaging examination is an important modality to evaluate the position and width of the tunnel. However, controversy remains regarding its value in evaluation. One of the controversies lies in the lack of a standard and reliable method to accurately define whether the femoral tunnel attachment is located at the normal anatomic site. Some authors proposed that femoral attachment of the reconstructed ligament could be assessed in a lateral X-ray^8^. However, we found that the attachments were usually not clearly visible on lateral radiographs. Furthermore, this method had a high demand for patient position. Thus, the application of X-ray is rather limited^9^. Some studies used MRI to measure the distance from tunnel to posterior aspect and to articular surface of femoral medial condyle^10^, however, the routine 3–5 mm thickness of MRI images leads to a partial volume effect. In addition, even slight artifact on MRI images caused by screws could affect the precise measurement of tunnel width and position due to the small tunnel diameter^11^.

Therefore, in this study, we used a new method based on CT examinations to evaluate the position and width of the femoral and patellar tunnel in patients who were followed up after patellar dislocation triple surgeries for more than one year, and further studied their influence on knee function.

## Materials and Methods

### Patients

Between January 2007 and July 2015, a series of 216 (254 knees) consecutive patients with recurrent patellar dislocation were admitted to our hospital. The inclusion criteria were as follows: (1) patients who were diagnosed with recurrent patellar dislocation clinically or radiologically; (2) patients who underwent patellar triple surgeries which were single-bundle MPFL reconstruction primarily, lateral retinaculum release and tibial tubercle osteotomy involving medializing and elevating the tubercle; 3) patients who had more than 1 year of follow-up and completed the knee function score table. The exclusion criteria were as follows: (1) patellar dislocation associated with surgery history or combined with rheumatoid arthritis, articular infection, or bone tumor; (2) trauma to the affected knee during the follow-up; (3) patients who were lost without completion of follow-up. In total, 66 patients (70 knees) were included in this study (male/female, 18/48; left/right knee 37/33; mean age, 24.3 years). All patients were examined by CT on the knee 3 days after procedures as baseline CT to evaluate patellofemoral joint, and then were examined again at more than 1 year, together with 2 sets of knee function score tables. We compared the baseline CT with the last CT, and determined the relationship between the findings of the last CT and knee function. This retrospective study was approved by our hospital’s ethics committee and the written informed consent was waived.

### Operative technique

Lumbar anesthesis; knee flexion at around 90°. Cut an oblique incision medial to tibial tubercle to take out gracilis tendon. Apply an arthroscopic check of knee joint via routine anterior-medial and anterior-lateral route, and of patellofemoral joint via the route medial-superior to patella, and then insert plasma knife anterior-laterally. Cut patellar lateral retinaculum ligament, and after the exposure of tibial tubercle, cut a piece of bone (measuring 6–8 cm) from it, move it medially and anteriorly by 1.5 cm at each direction, and stabilize it with titanium screw. Drill a tunnel with a 4.5 mm cannulated reamer at the upper 1/3 point of medial patella, and introduce one end of gracilis tendon into patellar tunnel. Drill another tunnel from femoral medial condyle with a 4.5 mm cannulated reamer to contralateral cortex, and overdrill the tunnel with a 8 mm cannulated reamer to a distance of 4 cm. Drag the gracilis tendon through the femoral tunnel and stabilize it by 7 mm-diameter absorbable screw at the contralateral cortex.

### Knee function evaluation

All patients were followed up at a minimum of 12 months. Objective postoperative knee function was assessed according to the Kujala score and Lysholm score, both of which included the evaluation of anterior knee pain, patellofemoral instability, redislocation, range of movement and level of activities. The two scores were each graded as excellent (>95), good (94–85), decent (84–65), or poor (<65). Patients were asked to fill in the two scoring system table at each follow-up.

### Radiological evaluation

**Figure 1:**
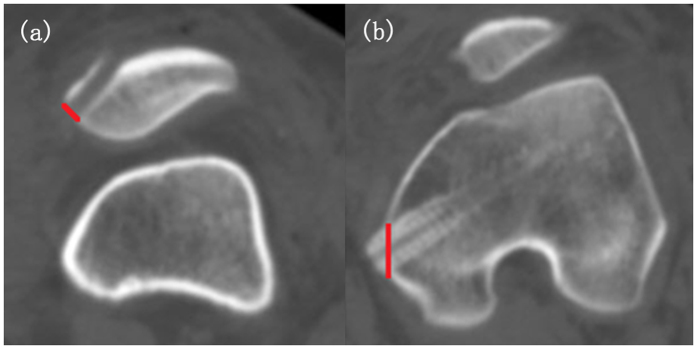
Methods of measuring width of bony tunnels after patellar dislocation triple-surgeries. (**a**) Solid line represents patellar tunnel width. (**b**) Solid line represents femoral tunnel width.

**Figure 2:**
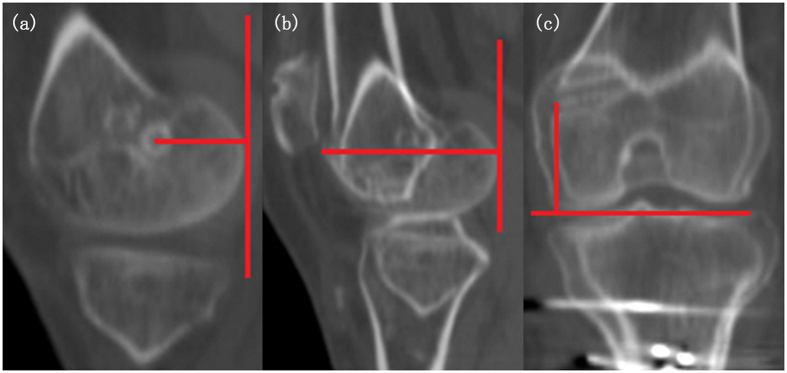
Method of measuring the location of bony tunnel after patellar dislocation triple-surgeries. (**a**) Imposed sagittal image which shows the tangent line of the most posterior aspect of medial condyle, as well as the distance between bony tunnel and posterior aspect of femoral medial condyle (BP). (**b**) Imposed sagittal image which shows the tangent line as same as in image 2.a and the distance between anterior and posterior aspect of medial condyle (AP). (**c**) Imposed coronal image which shows the tangent line of the most distal aspect of medial condyle and the distance between bony tunnel and distal aspect of medial condyle (BD).

All patients were arranged with a CT scan within one week after the completion of the function scoring tables at their latest follow-ups. The examination were performed using a 64-slice multidetector CT scanner (Lightspeed VCT 64, GE, Milwaukee, US) in the supine position with the knee extended. Scanning parameters were as follows: tube voltage was 120 kV, tube current was 180 mA, collimation was 64 × 0.625 mm, pitch was 0.516, rotation time was 1.0 s, thickness and interval were 0.625 mm. Scanning range extended from the level above patella to the level below tibial tuberosity. The data were then transferred to a post-process workstation (ADW4.3, GE) and picture archiving and communication system (PACS) for analysis. Two experienced radiologists who were blind to the clinical state of the patients independently reconstructed the images and measured the values. The methods of measurements were described as follows:

1. Patellar and femoral tunnel width: On axial images, the width of the patellar and femoral tunnel was determined by the maximum distance between the margins of cortex of the medial aspect of the patellar and femoral medial condyle, respectively (Fig. 1).
2. Position of the tunnel on the femoral medial condyle: First, reconstructed sagittal CT images showing the posterior aspect of femoral medial condyle (P), the anterior aspect of femoral medial condyle (A), and the entrance of bony tunnel (B) were obtained and then superposed with each other. We measured the distance between the anterior and posterior margin of the femoral medial condyle as reference, termed AP. Then, we measured the distance between the entrance of the bony tunnel and the posterior margin of the femoral medial condyle as BP. We described the position of the bony tunnel in the anterior-posterior direction as BP/AP × 100%, which was normally 40%. Furthermore, we obtained the reconstructed coronal CT images showing the distal aspect of medial condyle (D) and the entrance of the bony tunnel, and we superposed the two images. The distance between the two landmarks was designated as BD. We defined the position of bony tunnel in the proximal-distal direction as BD/AP × 100%, which was normally 50% (Fig. 2)^12^,^13^. For the assessment of position in the anterior-posterior direction, patients were divided into two groups with BP/AP ≤ 40% and BP/AP > 40%. For the assessment of position in the proximal-distal direction, they were separated into two groups with BD/AP ≤ 50% and BD/AP > 50%.
3. Patellofemoral measurements: Patellar tilt angle and patellar lateral displacement of all patients were measured^3^,^14^,^15^.
4. Patellar instability: TT-TG distance, Insall-Salvati (IS) index, and Dejour’s Trochlear Dysplasia Classification were included^16^,^17^,^18^. TT-TG distance represents distance between the lowest point of the trochlear groove and the tibial tubercle. IS is the ratio of the patellar length and the distance from the lowest point of the patella to the tibial tubercle.

### Statistical analysis

Statistical analysis was performed with statistical product and service solutions (SPSS) software package, version 22.0. The interobserver variability of all measurements was evaluated by intraclass correlation coefficient (*ICC*), which ranged from 0 to 1. *ICC* > 0.8 indicates excellent agreement, 0.61–0.8 indicates good agreement, 0.41–0.6 indicates decent agreement, and <0.41 indicates poor agreement. Paired student *t* tests were performed for the comparison of patellar and femoral tunnel width. The measurements were expressed as the mean ± standard deviation (SD). Correlative analysis of the bony tunnel position and knee function was performed with Pearson’s partial correlation coefficient. Patellar tilt angle, patellar lateral displacement, TT-TG distance, IS index and trochlear dysplasia classification were all selected as variables for Pearson’s partial correlation coefficient. *P* < 0.05 was defined as significant. Mann-Whitney U tests were performed for the comparison of knee function scores between patients with and without enlarged femoral tunnel.

## Results

In our study, follow-ups were performed at 12 months in 28 patients (29 knees) and after 12 months in 38 patients (41 knees), with average of 20.9 months (range, 12–78 months). No significant difference was found between the Kujala scores (mean, 81.8; range, 35–98) and Lysholm scores (mean, 83.5; range, 24–100) at the last follow up (*t* = −1.573, *P* = 0.120). One patient was diagnosed with persistent patellar dislocation, and two were diagnosed with transient patellar dislocation. At the last follow-up, 12 patients had trochlear dysplasia of grade A, and the others had trochlear dysplasia of grade B to D; 21 patients (22 knees) had TT-TG more than 20 mm, which was considered abnormal; 37 patients (40 knees) had IS larger than 1.2, which was diagnosed with patellar alta.

All measurements showed good or excellent interobserver variability (*ICC* = 0.765–0.877). BP/AP ranged from 21.11% to 72.12%, and BD/AP ranged from 18.31% to 69.43%. CT measurements of BP, AP and BD of the 4 different groups were summarized in Table 1. BP/AP was found to have a moderate positive correlation with the Kujala score in patient group with BP/AP ≤ 40%, and there was a moderate negative correlation with both the Kujala scores and Lysholm scores in patient group with BP/AP > 40%. However, no significant correlation was found between BD/AP and the Kujala or Lysholm score in all patients (Table 2).

Compared with the measurements obtained from the CT images taken 3 days after surgeries, both patellar and femoral tunnel width at the last CT examination enlarged significantly (Table 3). Femoral tunnel enlargement was noted in 54 knees and patellar tunnel enlargement in 41 knees. No significant difference in either Kujala score or Lysholm score was noted between patients with and without an enlarged femoral tunnel (Table 4).

## Discussion

In our study, the most significant findings were that we could use our method to evaluate the tunnel position of the reconstructed MPFL, and that the tunnel malposition was associated with impaired knee function during follow-up. Furthermore, changes, while would not affect knee function, were significant in terms of femoral and patellar tunnel width during the follow-up.

Compared with radiographs and MRI, the most significant benefit of CT is being able to reliably identify and quantify bony tunnel morphology, such as the cortical border and trochlear depth^19^,^20^. CT also possesses the merits of low requirement for patient position, a shorter scan time and multiplanar reconstruction (MPR) technique to readily display the detailed and comprehensive bone structures. On CT reconstructed sagittal images of knees, we applied the fomula for the “relative distance”, setting the distance from the anterior to the posterior femoral medial condyle as reference and adopted percentage to denote the location of the tunnel in two different orientations. We believed that relative distance could better eliminate the individual deviations related to age, sex, and trochlear dysplasia, which would greatly interfere with the accurate location of the tunnel. This method allowed us to locate tunnel of all patients on CT images. Because the reconstructed MPFL tunnel attachment should be located at its original anatomical position, we separated our patients based on BP/AP = 40% and BD/AP = 50% respectively to observe the relationship between the tunnel position and knee function^12^,^21^. In addition, some measurements representing patellar instability may indicate reduced knee function, so we included all these factors when performing the correlation analysis to objectively reflect the isolated impact of the tunnel attachment position on the recovery of knee function.

The tunnel location of the reconstructed MPFL is crucial to promote the postoperative recovery of knee function and to lower the possibility of complications such as recurrent dislocation, locking knee and knee pain. However, many controversies still remain regarding the relationship between these two factors. In a recent study, Hopper *et al*. reported that a good clinical outcome could be obtained only if the femoral position of reconstructed MPFL using an autograft was located within 10 mm of the normal attachment^22^. Stephen *et al*. showed that the position of reconstructed ligament attachment on the femur was more important in the proximal-distal direction than in the anterior-posterior position because even slight proximal or distal displacement of attachment was likely to give rise to the limitation of knee motion, increased pressure of medial patellofemoral joint and recurrent patellar dislocation^12^. On the contrary, both Servien *et al*. and Melegari *et al*. reported that they found no correlation between a malpositioned tunnel and knee function^10^,^23^.

In accordance with our study based on CT, when the position is abnormal in the anterior-posterior direction but not the proximal-distal direction, the postoperative recovery of knee function would be impaired. The reasons responsible for this phenomenon might be that a close proximity of the reconstructed MPFL to the patella would make the ligament too short to provide enough tension to prevent patellar lateral displacement, putting the MPFL in a constant state of tension during patellar displacement and leading to chronic ligament injury. Such conditions would give rise to recurrent patellar dislocations postoperatively. Secondly, a short graft associated with insufficient power could also lead to an uneven load on the patella, with a greater load on the medial aspect and a smaller load on the lateral aspect during the motion of the knee joint, eventually causing chronic fraying of the medial patellar cartilage. Besides, when the tunnel position is too posterior, the graft would be too long and lax to play an adequate role in preventing lateral patellar displacement during movement of the knee joint. Furthermore, a malpositioned tunnel would also result in increased intensity of the graft during flexion, leading to greater pressure on the patellofemoral joint, pain and weakened activity of the knee joint during flexion, as well as an elongated ligament^24^. There also have been several previous studies supporting our findings. Ntagiopoulos *et al*. reported that an anteriorly placed graft always resulted in a greater laterally directed load for translation during extension and a lower load during flexion than the original MPFL, vice versa^25^. Tateishi *et al*. found that when the graft was placed anteriorly or posteriorly, it resulted in poor restoration^26^. Studies by Oliveira *et al*. indicated that in patients with patellar instability, MPFL was approximately 30% longer and 50% thinner compared to that in an asymptomatic population^27^. It was estimated that the long MPFL itself represented patellar instability. Therefore, orthopaedists as well as radiologists should bear in mind that graft attachment must be in an anterior-posterior anatomic position during the procedures and postoperative evaluation. An improper graft position on CT is tended to be associated with a greater likelihood of patellofemoral pain, limitation of movement, and recurrent dislocation. For this reason, CT could provide valuable diagnostic information for the interpretation of clinical symptoms, detection of causes and further management in postoperative patients.

In addition, according to our study, compared with the follow-ups within 3 days after surgeries, we noted patellar and femoral tunnel enlargement after 12 months based on CT independent of the restoration of the knee joint. We estimated that tunnel enlargement may be caused by trochlear dysplasia, which was not treated during patellar dislocation triple surgery, as well as increased TT-TG and patellar alta that were still present in partial patients postoperatively. All these patellar instability factors that tended to put lateral stress on patella and screw could lead to tunnel enlargement. Berard *et al*. also found that within 3 years after MPFL reconstruction, radiographs in more than 40% of the patients demonstrated femoral tunnel enlargement that was not considered a risk factor for recurrent patellar dislocation postoperatively^28^. They estimated that patellar alta putting more force on the reconstructed ligament may cause tunnel enlargement. In addition, the process of ligamentisation after MPFL reconstruction may also be one of the causes of tunnel enlargement. ‘Ligamentisation’ which has been broadly recognized in ACL reconstruction refers to the process of an autograft converting into a ligament^29^. An autograft will undergo ‘ligamentisation’ for at least one year without sufficient function as a ‘normal’ ligament^30^. We surmised that the autograft might also had undergone such a process for one year after MPFL reconstruction and could not provide adequate force for preventing lateral patellar displacement as a native ligament, leading to micro-movement of the screw and secondary tunnel enlargement, particularly when patients undergo weight-bearing rehabilitation training that can overload the immature autograft. Several biological and mechanical factors also contribute to tunnel enlargement, such as synovial effusion including several toxic products, inflammatory response, absorbable screw and different characteristics of grafts^28^,^31^,^32^,^33^,^34^. On the basis of our results, we regarded tunnel enlargement as a normal physiological response of the cortex around tunnel to grafts, the surrounding tissue inflammation and joint activity.

Our study has some limitations. First, some patients were only followed up for a short or moderate duration. Whether chronic cartilage damage would occur in patients during long-term follow-up, leading to impairment of knee function, remains unknown and requires further research. Second, owing to the fear of recurrent dislocation, many patients in our study overlooked the importance of activity and did not perform sufficient restoration training to participate in sports activities, giving rise to a ‘pseudo-decrease or pseudo-increase’ of the subjective knee function scores. Finally, MRI examinations were not performed in our patients to detect combinative lesions, such as ligament injury, articular cavity adherence, and other soft tissue changes.

In conclusion, CT post-processing technique of superimposition could well display the position of femoral tunnel attachment, which had the greatest impact on patellofemoral function in anterior-posterior direction, and this could help radiologists and orthopaedists to more accurately assess the clinical outcomes of patellar dislocation triple surgeries.

## Additional Information

**How to cite this article:** Qin, L. *et al*. Relationship between bony tunnel and knee function in patients after patellar dislocation triple surgeries—a CT-based study. *Sci. Rep.* **7**, 41360; doi: 10.1038/srep41360 (2017).

**Publisher's note:** Springer Nature remains neutral with regard to jurisdictional claims in published maps and institutional affiliations.

## Figures and Tables

**Table 1:** Measurements of BP, AP and BD.

	BP/AP ≤ 40% (*n* = 31)	BP/AP > 40% (*n* = 39)	BD/AP ≤ 50% (*n* = 47)	BD/AP > 50% (*n* = 23)
BP (mm)	19.6 ± 3.8	30.2 ± 5.9	/	/
AP (mm)	59.8 ± 4.7	59.4 ± 4.5	60.2 ± 4.2	58.5 ± 5
BD (mm)	/	/	23.5 ± 4.4	32.9 ± 3.6

**Table 2:** Correlation between femoral tunnel position and knee function postoperatively.

	Kujala score		Lysholm score	
	*r*	*P* value	*r*	*P* value
BP/AP ≤ 40% (*n* = 31)	0.496	0.019	0.253	0.257
BP/AP > 40% (*n* = 39)	−0.483	0.006	−0.533	0.002
BD/AP ≤ 50% (*n* = 47)	−0.110	0.516	0.029	0.866
BD/AP > 50% (*n* = 23)	0.150	0.593	0.097	0.730

**Table 3:** Comparison of postoperative tunnel width.

	Within 3 days (*n* = 70)	Last follow-up (*n* = 70)	*t*	*P* Value
Femoral tunnel width (mm)	8.7 ± 2.3	10.6 ± 2.6	−6.102	<0.05
Patellar tunnel width (mm)	4.6 ± 1.1	5.1 ± 1.4	−2.760	0.007

**Table 4:** Comparison of patients with and without femoral tunnel enlargement at the last follow-up.

Patients	Kujala score	*P* Value	Lysholm score	*P* Value
Without femoral tunnel enlargement (*n* = 16)	79.4 ± 16.9	0.386	78.6 ± 18.4	0.085
With femoral tunnel enlargement (*n* = 54)	82.5 ± 10.4		84.8 ± 10	
